# Shared etiology of type 1 diabetes and Hashimoto’s thyroiditis: a population-based twin study

**DOI:** 10.1530/EJE-22-0025

**Published:** 2022-04-07

**Authors:** Jakob Skov, Ralf Kuja-Halkola, Patrik K E Magnusson, Soffia Gudbjörnsdottir, Olle Kämpe, Sophie Bensing

**Affiliations:** 1 Department of Molecular Medicine and Surgery, Karolinska Institutet, Stockholm, Sweden; 2 Department of Medicine, Karlstad Central Hospital, Karlstad, Sweden; 3 Department of Medical Epidemiology and Biostatistics, Karolinska Institutet, Stockholm, Sweden; 4 Institute of Medicine, University of Gothenburg, Sahlgrenska University Hospital, Gothenburg, Sweden; 5 National Diabetes Register, Centre of Registers, Gothenburg, Sweden; 6 Center for Molecular Medicine, Department of Medicine (Solna), Karolinska Institutet, Stockholm, Sweden; 7 Department of Endocrinology, Karolinska University Hospital, Stockholm, Sweden; 8 K.G. Jebsen Center for Autoimmune Diseases, University of Bergen, Bergen, Norway

## Abstract

**Objective:**

Type 1 diabetes and Hashimoto’s thyroiditis frequently cluster in individuals and in families, indicating shared origins. The objective of this study was to investigate familial co-aggregation of these diseases and to quantify shared genetic and environmental factors.

**Design:**

This study is a twin cohort study.

**Methods:**

National health registers were used to identify cases among 110 814 Swedish twins. Co-aggregation was calculated as risk ratios for type 1 diabetes among co-twins of individuals with Hashimoto’s thyroiditis, and vice-versa. Variance explained by genetics (i.e. heritability), and the proportions thereof shared between the diseases, was estimated by contrasting associations in monozygotic and dizygotic twins using structural equation models.

**Results:**

Individuals with one disease were at a high risk for the other disease (adjusted risk ratio: 11.4 (95% CI: 8.5–15.3)). Co-aggregation was more common in monozygotic than in dizygotic pairs, with adjusted risk ratios of 7.0 (95% CI: 3.2–15.1) and 1.7 (95% CI: 0.7–4.1), respectively. Genetic effects shared across diseases accounted for 11% of the variance for type 1 diabetes and 9% of the variance for Hashimoto’s thyroiditis, while environmental factors unique to individual twins, but shared across diseases, accounted for 10% of the variance for type 1 diabetes and 18% of the variance for Hashimoto’s thyroiditis.

**Conclusions:**

Both genes and environment unique to individual twins contribute to considerable etiologic overlap between type 1 diabetes and Hashimoto’s thyroiditis. These findings add to the current knowledge on the mechanisms behind autoimmune disease clustering and could guide future research aimed at identifying pathophysiological mechanisms and intervention targets.

## Introduction

Type 1 diabetes and hypothyroidism due to Hashimoto’s thyroiditis are two of the most common autoimmune diseases (1, 2). Co-occurrence of both diseases in the same individual is common, with up to one in four patients with type 1 diabetes harboring autoantibodies predisposing to thyroid disease (3) and more than one in ten eventually developing overt Hashimoto’s thyroiditis (4). Vice versa, the risk of type 1 diabetes is also increased in patients with Hashimoto’s thyroiditis, most notably in early onset disease (5). The combination of type 1 diabetes and Hashimoto’s thyroiditis in the same patient is in fact the most common autoimmune polyendocrinopathy, sometimes referred to as a variant of the autoimmune polyglandular syndrome type 3 (APS3). Moreover, familial studies have repeatedly demonstrated that relatives of individuals with one disorder are at increased risk of the other disorder, further supporting a common origin (6, 7, 8).

Both type 1 diabetes and Hashimoto’s thyroiditis are complex autoimmune disorders, with multiple genetic and non-genetic factors contributing to disease. Twin studies have established that genetic components (i.e. heritability) explain most of the observed variance for both diseases (9, 10, 11), and molecular genetic studies have identified genetic polymorphisms linked to both disorders (12). Still, monozygotic twin pairs affected by either disease are usually discordant, with only one twin affected by the disease, underscoring the importance of environmental factors in triggering autoimmunity (13). Hence, the magnitude and make-up of the shared origin of these diseases are still largely unknown.

The aim of this study was to assess shared familial risk across type 1 diabetes and Hashimoto’s thyroiditis and to quantify common genetic and environmental origins of disease using a large, population-based cohort of twins.

## Subjects and methods

### Data sources and study population

The personal identity number, a unique identifier assigned to all Swedish residents (>99.9%) at birth or upon immigration, is used for all health care contacts. It thus allows for cross-register linkages with essentially perfect coverage (14).

The Swedish Twin Registry (STR) contains information on twins born in Sweden from the late 19th century onwards, with a coverage of approximately 95% for birth years relevant to this study. Zygosity has been determined according to a validated intra-pair similarity algorithm, DNA, and opposite sex, in approximately 90% of twin pairs born before 1959 and in 70–80% of most, but not all, later birth-cohorts (15). The study population encompassed all twins of known zygosity, born up until December 31, 2006, with information on both twins in a pair present in the STR. To maximize power, information on both same-sex and opposite-sex dizygotic twin pairs was included, and to improve coverage and diagnostic precision, both twins in a pair were required to be alive (or not yet born) in 1976 for inclusion. A total of 120 286 individuals from complete twin pairs were identified in the STR. Of these, 3966 were excluded because of unknown zygosity. A further 3453 individuals deceased before 1976 were excluded along with 2049 co-twins of deceased twins. Two twin pairs with ambiguous birth data were also excluded, yielding a final sample of 110 814 twins.

The National Diabetes Register, which holds data on nearly all individuals with diabetes in Sweden, was used to identify diagnoses of type 1 diabetes through 2015. Type 1 diabetes is defined in the register on the basis of treatment with insulin only and a diagnosis at age 30 years or younger, and has been validated as accurate, with a positive predictive value of 97% (16).

The National Patient Register (NPR) (17) and the Prescribed Drug Register (PDR) (18) were used to identify cases of overt Hashimoto’s thyroiditis. The NPR, found in 1964, holds inpatient discharge records, coded according to the International Statistical Classification of Diseases. The NPR reached nationwide coverage (>99%) in 1987. As of 2001, records for hospital-associated outpatient care are also included in the register. The PDR, in operation since 2005, collects data on all prescribed drugs dispensed in Sweden, coded according to the Anatomical Therapeutic Chemical Classification System (ATC).

In brief, Hashimoto’s thyroiditis was defined as an ICD diagnosis of Hashimoto’s thyroiditis, without records suggesting congenital, drug-induced, infectious, postprocedural, or iodine-deficient hypothyroidism. For patients alive in 2006, multiple (≥2) dispensations of levothyroxine (ATC H03AA) were required to validate an ICD diagnosis of Hashimoto’s thyroiditis. Importantly, in individuals with type 1 diabetes, multiple (≥2) dispensations of levothyroxine were considered sufficient for a diagnosis of Hashimoto’s thyroiditis in the absence of ICD diagnoses indicating other thyroid disorders, as Hashimoto’s thyroiditis is sometimes not ICD-coded when co-occurring with type 1 diabetes (diagnostic criteria are detailed in Supplementary Table 1, see section on supplementary materials given at the end of this article).

### Statistical analysis

Type 1 diabetes and Hashimoto’s thyroiditis were defined as binary variables indicating the presence or absence of disease prior to end of follow-up. In a subanalysis, we also examined the APS3 phenotype.

#### Association and (co-)aggregation

Disease associations were estimated as risk ratios, that is the ratio of probabilities of Hashimoto’s thyroiditis among individuals with, compared with individuals without, type 1 diabetes. Familial aggregations were calculated as the risk of disease among individuals whose co-twin had the same disease compared with individuals whose co-twin did not have the same disease. We estimated familial co-aggregation as risk ratios for type 1 diabetes among individuals whose co-twin had Hashimoto’s thyroiditis compared to individuals whose co-twin did not have the disease (and *vice versa*). We estimated aggregation and co-aggregation separately by zygosity. All estimates were calculated as crude and adjusted for sex and birth cohort (<1940, 1940–1959, 1960–1979, and >1979). For these analyses, we used generalized estimating equations with log-link and standard errors clustered on twin pair ID.

#### Concordances and tetrachoric correlations

A twin pair is considered concordant affected for a trait when both twins are affected and discordant when only one twin is affected. A higher concordance in monozygotic than in dizygotic twins indicates a genetic contribution. Probandwise concordance rates were therefore calculated separately by zygosity to give an absolute measure of disease risk. We also calculated concordance rates across disorders, that is the proportion of individuals diagnosed with one disease whose co-twin had the other disease and *vice versa*.

Next, tetrachoric correlations, a measure of associations between dichotomous traits (e.g. disease or no disease), were estimated. For tetrachoric correlations, an un-observed normally distributed liability for the disease is assumed to exist. The disease becomes manifest when an individual exceeds a threshold on this assumed disease liability, and this threshold is estimated from the data. When assessing dichotomous disease in the two twins in a pair, the observed 2-by-2-table of disease vs no disease in one twin cross-tabulated with his/her co-twin is assumed to reflect a combination of the two normally distributed liabilities in the two twins (with the same thresholds as mentioned above). The distribution of concordance and discordance in the 2-by-2-table of disease across the twins allows the correlation between the liabilities to be inferred – this correlation is what is referred to as the tetrachoric correlation. A higher tetrachoric correlation in monozygotic than in dizygotic twin pairs is indicative of genetic influences on a trait. This measure is also the basis of subsequent calculations of heritability. We estimated tetrachoric correlations within individuals across disorders, referred to as phenotypic correlation, between twins in pairs within the same disorder, referred to as intra-class correlations (ICC), and between twins in pairs across disorders, referred to as cross-twin cross-trait correlations (CTCT). Tetrachoric correlations were estimated using structural equation models, unadjusted and adjusted for birth cohort and sex.

#### Quantitative genetic modeling

Quantitative genetic analyses were based on the classic twin assumptions that monozygotic twins are genetically identical, that dizygotic twins share on average half of their segregating alleles, and that monozygotic and dizygotic twins share environment to a similar degree. Bivariate quantitative genetic models were fitted in a structural equation modeling framework. In this model, the variance within disorders and covariance between disorders were modeled to result from additive genetic factors (A; the heritability), dominance deviations (D), shared environmental factors affecting both twins in a pair (C), and unique environmental factors not shared by twins (E). ACE (including A, C, and E), ADE, AE, and CE models were fitted by estimating A, D, C, and E contributions to each disorder, as well as the broad-sense heritability (H; the sum of A and D), when applicable. We also estimated the correlation between these sources of variance, that is the genetic correlation, r_A_, and r_D_, r_C_, and r_E_, as well as a broad sense genetic correlation, r_H_. Next, we calculated the inferred proportion of explained variance from A, D, C, and E, as well as H in type 1 diabetes shared with Hashimoto’s thyroiditis and *vice versa*. Model fit was compared using Akaike information criterion (AIC) and Bayesian information criterion (BIC), where a lower value indicates a better fitting model.

#### Subanalysis

We defined APS3 as present in individuals diagnosed with both type 1 diabetes and Hashimoto’s thyroiditis and absent in all other individuals. We estimated concordance rates and tetrachoric correlations and fitted quantitative genetic models for this variable.

For statistical analyses, we used R (R-Development-Core-Team, 2010) with packages drgee (19), and OpenMx (20).

This study was approved by the Regional Ethical Review Board in Stockholm, Sweden (Dnr: 2017/1546-32). Informed consent was waived by the ethics committee.

## Results

### Descriptive

Overall, 364 individuals (179 women, 185 men) had type 1 diabetes and 1683 individuals (1410 women, 273 men) fulfilled the diagnostic criteria for Hashimoto’s thyroiditis. Co-occurrence of both diseases was found in 48 individuals (37 females, 11 males), with 13.2% (48/364) of patients with type 1 diabetes also having Hashimoto’s thyroiditis, and 2.8% (48/1683) of patients with Hashimoto’s thyroiditis also diagnosed with type 1 diabetes. Disease prevalence according to year of birth, sex, and zygosity is presented in Table 1. Age at diagnosis and year of diagnosis are outlined in Supplementary Fig. 1.

**Table 1:** Descriptive, number of individuals, and column percent. Data are presented as *n* (%).

	Total (%)	T1D (%)^a^	HT (%)^a^	T1D and HT (%)^a^
Total	110 814 (100.0)	364 (0.3)	1,683 (1.5)	48 (<0.1)
Sex				
Males	52 171 (47.1)	185 (50.8)	273 (16.2)	11 (22.9)
Females	58 643 (52.9)	179 (49.2)	1,410 (83.8)	37 (77.1)
Birth year				
<1940	30 190 (27.2)	27 (7.4)	843 (50.1)	3 (6.2)
1940–1959	28 948 (26.1)	118 (32.4)	509 (30.2)	13 (27.1)
1960–1979	14 082 (12.7)	101 (27.7)	189 (11.2)	17 (35.4)
>1979	37 594 (33.9)	118 (32.4)	142 (8.4)	15 (31.2)
Zygosity				
Monozygotic	35 990 (32.5)	116 (31.9)	553 (32.9)	22 (45.8)
Dizygotic	74 824 (67.5)	248 (68.1)	1,130 (67.1)	26 (54.2)

^a^Categories not mutually exclusive.

HT, Hashimoto’s thyroiditis; T1D, type 1 diabetes.

### Association and (co-)aggregation

Results for association and (co-)aggregation are presented in Table 2. The adjusted risk ratio (aRR) for co-occurrence of both diseases (within individual association) was 11.4 (95% CI: 8.5–15.3). Disease aggregation (same disease in co-twins) was present in both monozygotic and dizygotic twins, with risk ratios higher for type 1 diabetes than for Hashimoto’s thyroiditis, possibly reflecting a lower population prevalence for type 1 diabetes. The highest relative risk was seen in monozygotic pairs, with aRR 131.3 (95% CI: 80.1–215.2) for type 1 diabetes and 14.5 (95% CI: 11.1–18.9) for Hashimoto’s thyroiditis, but aggregation was also statistically significant in dizygotic twin pairs (type 1 diabetes aRR: 11.8 (95% CI: 5.3–26.4); Hashimoto’s thyroiditis aRR 4.9 (95% CI: 3.8–6.4)). Co-aggregation, defined as risk ratios for Hashimoto’s thyroiditis among individuals whose co-twin had type 1 diabetes, was stronger in monozygotic (aRR: 7.0 (95% CI: 3.2–15.1)) than dizygotic twin pairs (aRR: 1.7 (95% CI: 0.7–4.1)). Results were similar when switching diseases in terms of exposure and outcome (Supplementary Table 2).

**Table 2:** Association, familial aggregation, and familial co-aggregation of type 1 diabetes and Hashimoto’s thyroiditis in twins.

	RR (95% CI)	aRR^a^ (95% CI)
Within disorders		
Risk of T1D when co-twin has T1D		
Monozygotic twins	175.5 (110.6–278.6)	131.3 (80.1–215.2)
Dizygotic twins	15.3 (6.9–34.1)	11.8 (5.3–26.4)
Risk of HT when co-twin has HT		
Monozygotic twins	26.6 (21.1–33.4)	14.5 (11.1–18.9)
Dizygotic twins	7.3 (5.6–9.5)	4.9 (3.8–6.4)
Between disorders^b^		
Risk of HT in individuals with T1D	8.9 (6.7–11.9)	11.4 (8.5–15.3)
Risk of HT when co-twin has T1D		
Monozygotic twins	6.3 (3.0–13.0)	7.0 (3.2–15.1)
Dizygotic twins	1.3 (0.6–3.2)	1.7 (0.7–4.1)

^a^Adjusted for sex and birth cohort; ^b^T1D was considered exposure and HT outcome. Results from analyses using HT as exposure and T1D as outcome are found in Supplementary Table 2.

HT, Hashimoto’s thyroiditis; T1D, type 1 diabetes;.

### Concordances and tetrachoric correlations

Type 1 diabetes was present in 337 twin pairs and Hashimoto’s thyroiditis in 1545 pairs. 27 pairs were concordant for type 1 diabetes and 138 for Hashimoto’s thyroiditis with 16 twin pairs demonstrating cross-twin-cross-trait concordance (different diseases in co-twins). For all disease combinations, concordance rates were higher in monozygotic than in dizygotic pairs, resulting in higher tetrachoric correlations (Table 3).

**Table 3:** Concordances, discordances, and tetrachoric correlations for type 1 diabetes and Hashimoto’s thyroiditis.

Within disorders					
	Concordant non-affected	Discordant	Concordant affected	Concordance rate	Tetrachoric correlations^a^, adjusted^b^
Type 1 diabetes					
MZ pairs	17 900	74	21	0.36 (0.27–0.49)	0.82 (0.75–0.89)
DZ pairs	37 170	236	6	0.05 (0.02–0.10)	0.37 (0.23–0.51)
Hashimoto’s thyroiditis					
MZ pairs	17 523	391	81	0.29 (0.25–0.35)	0.66 (0.61–0.71)
DZ pairs	36 339	1016	57	0.10 (0.08–0.13)	0.35 (0.29–0.42)
From type 1 diabetes to Hashimoto’s thyroiditis					
	Neither disease	T1D only	T1D and HT	Proportion with T1D and HT	Tetrachoric correlations^a^, adjusted^b^
Individuals	108 815	316	48	0.13 (0.10–0.17)	0.45 (0.39–0.51)
MZ pairs	35 332	105	11	0.09 (0.05–0.20)	0.28 (0.17–0.39)
DZ pairs		243	5	0.02 (0.01–0.05)	0.08 (-0.03–0.20)
From Hashimoto’s thyroiditis to type 1 diabetes					
	Neither disease	HT only	T1D and HT	Proportion with T1D and HT	Tetrachoric correlations^a^, adjusted^b^
Individuals	108 815	1635	48	0.03 (0.02–0.04)	0.45 (0.39–0.51)
MZ pairs	35 332	542	11	0.02 (0.01–0.04)	0.28 (0.17–0.39)
DZ pairs	73 451	1125	5	0.00 (0.00–0.01)	0.08 (-0.03–0.20)

^a^For tetrachoric correlations across T1D and HT, the correlation is the phenotypic correlation, and no directionality exists, i.e. estimates are exactly the same in ‘From T1D to HT’ and ‘From HT to T1D’ sections; ^b^Adjusted for sex and birth cohort.

HT, Hashimoto’s thyroiditis; T1D, type 1 diabetes;.

### Quantitative genetic modelling

**Figure 1:**
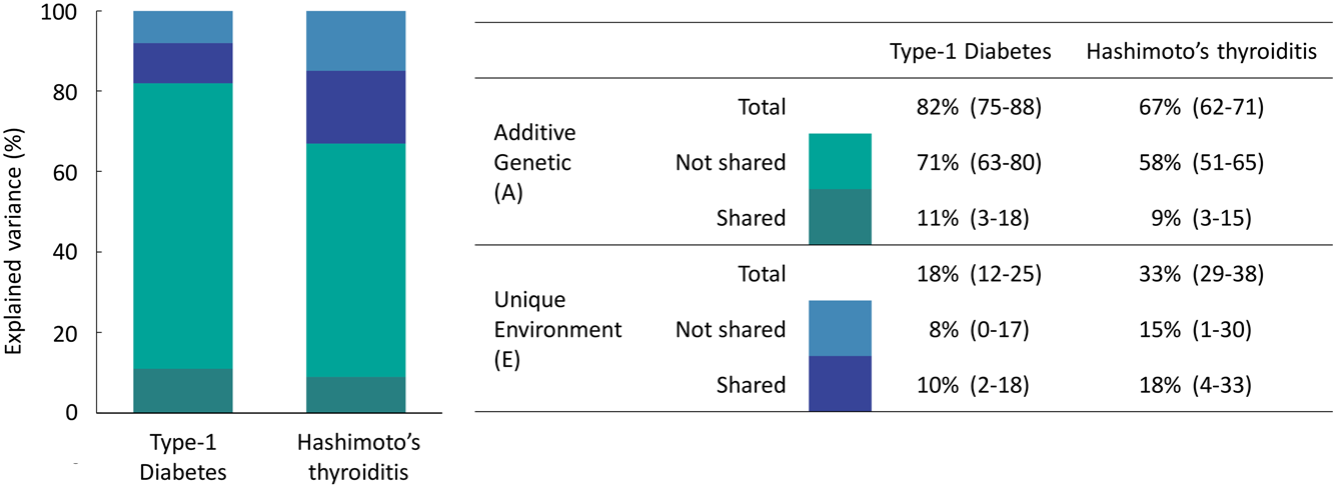
A, additive genetic effects. E, environmental effects not shared by co-twins. A is equivalent to heritability. Estimates adjusted for sex and birth cohort.

Overall, the AE model, with components of variance attributed to additive genetic factors (A) and unique environmental factors (E), provided the best fit according to both AIC and BIC, thus we present results from the adjusted AE model (Table 4), with results of the full models detailed in Supplementary Tables 3 and 4. Additive genetic factors accounted for 82% (95% CI: 75–88) of variance for type 1 diabetes and 67% (95% CI: 62–71) of variance for Hashimoto’s thyroiditis. The phenotypic correlation between the two diseases was 0.45 (95% CI: 0.39–0.50), with additive genetic sources (bivariate heritability) accounting for 59% (95% CI: 44–77) of the covariation and unique environment explaining 41% (95% CI: 23–59). The additive genetic (r_A_) and unique environmental correlations (r_E_) were 0.36 (95% CI: 0.23–0.48) and 0.74 (95% CI: 0.45–1.00), respectively (Table 4). Additive genetic factors shared between disorders explained 11% (95% CI, 3–18) of the variation in type 1 diabetes and 9% (95% CI, 3–15) in Hashimoto’s thyroiditis. The corresponding values for environmental factors shared between diseases but not between individuals was 10% (95% CI: 2–18) for type 1 diabetes and 18% (95% CI: 4–33) for Hashimoto’s thyroiditis (Fig. 1).

**Table 4:** Adjusted results of the best fitting AE-model^a^.

Univariate estimates	
Type 1 diabetes	
A	82% (75–88)
E	18% (12–25)
Hashimoto’s thyroiditis	
A	67% (62–71)
E	33% (29–38)
Bivariate estimates	
Phenotypic correlation	0.45 (0.39–0.50)
r_A_	0.36 (0.23–0.48)
r_H_	0.36 (0.23–0.48)
r_E_	0.74 (0.45–1.00)
Bivariate A	0.59 (0.41–0.77)
Bivariate H	0.59 (0.41–0.77)
Bivariate E	0.41 (0.23–0.59)

A, variance explained by additive genetics/narrow sense heritability; E, variance explained by individually unique environment; r_A_, correlation between additive genetic variance components for type 1 diabetes and Hashimoto’s thyroiditis (similar for r_E_); Bivariate A, explained phenotypic correlation by A sources of (co)variance (similar for Bivariate E). CIs are of Wald-type, thus they may span outside the parameter space but have been truncated at the parameter bound; ^a^Adjusted for sex and birth cohort.

### Subanalysis

The APS3 phenotype was rare with a total of 48 twins (37 women, 11 men) fulfilling diagnostic criteria. All 26 dizygotic pairs were discordant for APS3. Among 18 monozygotic pairs affected, 4 were concordant and 14 discordant, yielding a monozygotic concordance rate of 0.36 (0.18–0.74) and an estimated heritability of 0.85 (0.71–0.98) according to the preferred AE-model (Supplementary Tables 5 and 6).

## Conclusions

In this population-based study of Swedish twins, we found evidence of considerable etiologic overlap between type 1 diabetes and Hashimoto’s thyroiditis. Genetic effects were correlated across the diseases, with 11% (type 1 diabetes) and 9% (Hashimoto’s thyroiditis) of variance in each disease explained by additive genetic effects common to both disorders. Additionally, environmental factors unique to individual twins, but shared across diseases, accounted for 10% (type 1 diabetes) and 18% (Hashimoto’s thyroiditis) of variance. To the best of our knowledge, this is the first study to quantify shared etiologic fractions contributing to these disorders.

Co-occurrence of type 1 diabetes and Hashimoto’s thyroiditis was common among individual twins (aRR 11.4), and the prevalence of Hashimoto’s thyroiditis among patients diagnosed with type 1 diabetes was high at 13.2%, consistent with a prevalence of 9.8% reported in a recent meta-analysis (4). Conversely, type 1 diabetes was slightly more prevalent among individuals with Hashimoto’s thyroiditis (2.9%) than estimates of around 1% based on other European cohorts (8, 21). In line with previous studies, additive genetic factors (i.e. heritability) accounted for most of the phenotypic variance in both disorders, with unique environments explaining a smaller proportion (9, 10, 11, 22). Co-aggregation of diseases was also apparent, albeit statistically significant only among monozygotic twin pairs. This was reflected by a phenotypic correlation of 0.45, with substantial genetic correlation (0.36) and unique environment correlation (0.74) across diseases. In fact, the genetic coherence across diseases was similar in magnitude to the genetic overlap between Hashimoto’s thyroiditis and Graves’ disease, with 11% of variance across autoimmune thyroid diseases explained by common gene variants (22).

Several genetic polymorphisms have been implicated in both type 1 diabetes and Hashimoto’s thyroiditis (23), but results are inconsistent across different ethnicities, and varying definitions or thyroid autoimmunity, or lumping of Hashimoto’s thyroiditis and Graves’ disease into autoimmune thyroid disease, complicate interpretations (24). Focusing on genetic liability for type 1 diabetes and either overt Hashimoto’s thyroiditis or autoantibodies associated with Hashimoto’s thyroiditis in Caucasian populations narrows the list to only a few gene variants. These include HLA-DR3 and DR4 in association with DQ2 and DQ8 (25) and alleles of *PTPN22* (24, 26, 27) and *CTLA-4* (28, 29). Collectively, the identified polymorphisms explain only a small part of the genetic overlap between type-1 diabetes and Hashimoto’s thyroiditis reported in this study, suggesting other shared loci remain to be identified. There is also some evidence to support that APS3 represents a genetically distinct phenotype, with polymorphisms conferring risk for APS3 alone but not for isolated disease (30, 31). The APS3-variant was uncommon among our twins but demonstrated a high heritability of 85%, in line with estimates for organ-specific (single) autoimmune diseases (32).

The influence of unique environmental factors accounted for modest proportions of variance for type 1 diabetes and Hashimoto’s thyroiditis. Nevertheless, the environmental overlap was large, with 10–18% of the variance attributable to factors shared across the disorders but unique to individuals. Our findings contrast with results from a co-twin control analysis by Wang *et al.* (33) that did not find evidence of association between type 1 diabetes and thyroid peroxidase autoantibodies (TPOab) over and above shared factors in twin pairs (i.e. shared genetic and environmental influences). This may reflect differences in the etiology of TPOab vs overt HT, underlying differences between the examined populations or insufficient power to detect an association in the study by Wang due to a smaller twin sample.

In light of the dramatic increase in the incidence of autoimmune diseases in recent decades, including type 1 diabetes and Hashimoto’s thyroiditis (34, 35), environmental overlap should perhaps not come as a surprise. Rapid changes in disease incidences typically reflect environmental rather than genetic effects, and emergence of some environmental triggers shared across several autoimmune diseases, rather than a multitude of triggers unique to individual disorders, seems reasonable.

The hygiene hypothesis, stating that a lack of early life microbial exposure can increase susceptibility to immune-mediated diseases, is supported by studies on type 1 diabetes and early stages of thyroid autoimmunity. These demonstrate large differences in disease prevalence in genetically similar populations exposed to different environments (36, 37). However, with living conditions typically similar for co-twins, hygiene is best suited for explaining shared environmental components (C), which we did not detect. Nevertheless, some infections have been shown to increase the risk of type 1 diabetes and Hashimoto’s thyroiditis (38, 39), consistent with unique environmental exposure (E), but no single pathogen has been linked to both diseases. In fact, with the exception of smoking, which is known to reduce the risk of Hashimoto’s thyroiditis (39), and parental smoking during pregnancy, which has been linked to a reduced risk of type 1 diabetes in offspring (40), no unique environmental factors have been shown to alter the risk of both type 1 diabetes and Hashimoto’s thyroiditis in the same direction. Environmental triggers linked to type 1 diabetes are typically encountered early in life. These include high birth weight, early life dietary patterns, and enteroviral or respiratory infections at a young age (38). Interestingly, the observed overlap in unique environment suggests that early life or prenatal factors may be important triggers in Hashimoto’s thyroiditis as well. The influence of birth weight and prematurity on thyroid autoimmunity has indeed been studied, with mixed results (41, 42), while a small but significant increase in risk of Hashimoto’s thyroiditis in individuals born in summer, a potential proxy for exposure to seasonal infections early in life, has been demonstrated (43).

A major strength of this study was the use of nationwide registers with a high coverage, enabling us to examine a majority of Swedish twins, thus limiting selection bias. Moreover, the long observation period allowed us to detect most cases of two autoimmune diseases that typically debut decades apart. This was especially important for Hashimoto’s thyroiditis, lack of biochemical variables, most notably serologic data confirming autoimmune etiology, but also hormone levels, is a limitation to this study. However, the definition of type 1 diabetes in the National Diabetes Register has demonstrated high validity, with a positive predictive value of 97% (16). The validity of diagnostic records in the NPR for HT has not been evaluated, but a Danish study using virtually identical ICD and ATC codes reported a misclassification of <2% when compared with clinical records (44). With changes in register coverage over time, a first diagnostic record of Hashimoto’s thyroiditis may not reflect the date of diagnosis. We therefore refrained from running time-to-event analyses. We also lacked information on lifestyle factors including smoking and alcohol. Moreover, we used slightly different inclusion criteria for Hashimoto’s thyroiditis in subjects with type 1 diabetes compared to individuals without diabetes, which could potentially have inflated estimates of environments unique to the individual (E) but shared between the diseases. In addition, if within-individual co-occurrence of diseases was more likely to be detected; due to closer clinical surveillance in individuals with a prior diagnosis as compared to healthy individuals (i.e. screening for Hashimoto’s thyroiditis in patients with type 1 diabetes), this could have biased our results. The same would be true if monozygotic twins were more often screened for disease present in their co-twin, as compared to dizygotic twins. Finally, despite using a large twin sample, we did not have sufficient data to explore shared origins to type 1 diabetes and Graves’ disease, and based on previous findings that Graves’ disease and Hashimoto’s thyroiditis appear to be etiologically distinct, we chose not to cluster them into a combined autoimmune thyroid disease phenotype (22).

In summary, this is the first study to quantify the shared etiology between type 1 diabetes and Hashimoto’s thyroiditis. Our findings expand the current knowledge by demonstrating a considerable etiologic overlap, explained by genetic, but also individually unique environmental factors. This provides a foundation for future research aimed at characterizing the underlying biological mechanisms and modifiable risk factors.

## Supplementary materials

Supplementary Material

## Declaration of interest

The authors declare that there is no conflict of interest that could be perceived as prejudicing the impartiality of this study.

## Funding

County Council of Värmland (J S), the regional agreement on medical training and clinical research (ALF) between Stockholm County Council and Karolinska Institutet (S B), Swedish Society for Medical Research (S B), Åke Wiberg Foundation (S B), The Swedish Research Council (O K), and Novo Nordisk Foundation (O K).

## Author contribution statement

J S, R K-H, O K, and S B conceived the study. J S and R K-H designed the study and wrote the first draft of the manuscript. R K-H carried out statistical analysis. P M and S G acquired the necessary data. All authors were involved in the revision of the manuscript and approved the final version. J S and R K-H are the guarantors of this work and, as such, had full access to all of the data in the study and take responsibility for the integrity of the data and the accuracy of the data analysis.
