# Supplementary material for: Shared etiology of type 1 diabetes and Hashimoto’s thyroiditis: a population-based twin study
Source: Eur J Endocrinol. 2022 Apr 7;186(6):677–85. doi: 10.1530/EJE-22-0025 (PMC9175555; doi:10.1530/EJE-22-0025)

Supplementary material

Supplementary Table 1. Diagnostic criteria

<table>
<tr><td colspan="5">Hashimotos thyroiditis</td></tr>
<tr><td></td><td>ICD10</td><td>ICD9</td><td>ICD8</td><td>ICD7</td></tr>
<tr><td rowspan="2">Inclusion criteria</td><td>E035
E039
E063
E065</td><td>244X
245C
245W</td><td>244,0
245,03</td><td>253,00
253,19
253,29
254,00</td></tr>
<tr><td colspan="4">Or:
A diagnosis of Addison’s disease or type-1 diabetes and at least two filled prescriptions for levothyroxine (ATC-H03AA)</td></tr>
<tr><td rowspan="2">Exclusion criteria</td><td>E030
E031
E032
E033
E050
E055
E064
E890
E00
E01
E02
E060
E061</td><td>242A
243
244A
244B
244C
244D
244W
246B</td><td>242,00
242,09
243,99
245,00
245,01
245,02
245,04
244,00</td><td>057,11
252,00
252,01
252,02</td></tr>
<tr><td colspan="4">Or:
procedural codes
BAA60, BAA40, BAA50, BAA20
810, 811, 814, 820, 812, 813, 822, 824, 830

Or:
If alive 1 jan 2006; failure to fill at least two prescription for levothyroxine (ATC- H03AA)</td></tr>
<tr><td colspan="5">Type 1 diabetes</td></tr>
<tr><td>Inclusion criteria</td><td colspan="4">Included in the National Diabetes Register with a diagnosis of type-1 diabetes at age 30 years or younger, and treated with insulin only.</td></tr>
<tr><td>Exclusion criteria</td><td colspan="4">None</td></tr>
</table>

Supplementary Table 2. Association and familial co-aggregation between type 1 diabetes and Hashimoto's thyroiditis.

	Crude risk ratio	Adjusted[a] risk ratio
**Between disorders[b]**		
Within individual association	9.9 (7.2-13.6)	13.6 (9.6-19.3)
Co-aggregation monozygotic	6.7 (3.1-14.8)	8.0 (3.4-18.9)
Co-aggregation dizygotic	1.3 (0.6-3.2)	1.8 (0.7-4.3)

[a] Adjusted for sex and birth cohort

[b] Hashimoto's thyroiditis was considered exposure and Type 1 diabetes outcome.

Supplementary Table 3. Quantitative genetic results, crude models.

	ACE	ADE	**AE**	CE
AIC	-443,224.70	-443,223.09	**-443,228.84**	-443,142.79
BIC	-2,420,611.63	-2,420,610.02	**-2,420,642.54**	-2,420,556.49
		Univariate estimates		
T1D				
A	81% (50-100)	69% (12-100)	**83% (76-89)**	NA
D	NA	15% (0-73)	**NA**	NA
H	81% (50-100)	83% (77-90)	**83% (76-89)**	NA
C	2% (0-31)	NA	**NA**	74% (68-80)
E	17% (10-24)	17% (10-23)	**17% (11-24)**	26% (20-32)
HT				
A	60% (45-76)	70% (45-95)	**71% (66-75)**	NA
D	NA	0 (0-27)	**NA**	NA
H	60% (45-76)	71% (65-76)	**71% (66-75)**	NA
C	9% (0-22)	NA	**NA**	57% (53-61)
E	31% (26-36)	29% (24-35)	**29% (25-34)**	43% (39-47)
		Bivariate estimates		
Phenotypic correlation	0.37 (0.31-0.43)	0.34 (-0.18-0.87)	**0.37 (0.31-0.43)**	0.34 (0.29-0.40)
r_A	0.35 (-0.11-0.82)	0.26 (-0.45-0.97)	**0.26 (0.13-0.40)**	NA
r_D	NA	1.00 (-1.00-1.00)	**NA**	NA
r_H	0.35 (-0.11-0.82)	0.27 (0.12-0.41)	**0.26 (0.13-0.40)**	NA
r_C	-1.00 (-1.00-1.00)	NA	**NA**	0.14 (0.02-0.27)
r_E	0.71 (0.34-1.00)	0.73 (0.35-1.00)	**0.73 (0.38-1.00)**	0.75 (0.54-0.96)
Bivariate A	67% (-20-154)	53% (-15-120)	**55% (31-80)**	NA
Bivariate D	NA	7% (-160-175)	**NA**	NA
Bivariate H	67% (-20-154)	60% (-49-169)	**55% (31-80)**	NA
Bivariate C	-12% (-84-61)	NA	**NA**	27% (4-49)
Bivariate E	45% (18-72)	47% (-20-115)	**45% (20-69)**	73% (51-96)

AIC, Akaike's information criterion; BIC, Bayesian information criterion; T1D, type 1 diabetes; HT, Hashimoto's thyroiditis; A, additive genetics/narrow sense heritability; D, dominance deviations; H, broad sense heritability (A+D); C, shared environment; E, individually unique environment; ACE, ADE, AE, CE, models containing named sources of (co)variance; NA, not applicable; Bivariate A, explained phenotypic correlation by A sources of (co)variance (similar for, H, C, and E).

Confidence intervals are of Wald-type, thus they may span outside the parameter space, when this happened the interval has been truncated at the parameter bound. **Bold** font indicates best fitting model according to AIC and BIC.

Supplementary table 4. Quantitative genetic results, adjusted[a] models

	ACE	ADE	**AE**	CE
AIC	-443,178.04	-443,177.70	**-443,183.30**	-443,097.79
BIC	-2,420,493.59	-2,420,493.25	**-2,420,525.62**	-2,420,440.11
Univariate estimates				
T1D				
A	80% (50-100)	65% (9-100)	**82% (75-88)**	NA
D	NA	18% (0-74)	**NA**	NA
H	80% (50-100)	82% (76-89)	**82% (75-88)**	NA
C	1% (0-30)	NA	**NA**	72% (66-78)
E	18% (11-25)	18% (11-24)	**18% (12-25)**	28% (22-34)
HT				
A	0.60 (0.44-0.76)	0.66 (0.40-0.91)	**67% (62-71)**	NA
D	NA	0.01 (0.00-0.28)	**NA**	NA
H	0.60 (0.44-0.76)	0.67 (0.61-0.72)	**67% (62-71)**	NA
C	0.06 (0.00-0.19)	NA	**NA**	53% (49-57)
E	0.34 (0.29-0.39)	0.33 (0.28-0.39)	**33% (29-38)**	47% (43-51)
Bivariate estimates				
Phenotypic correlation	0.45 (0.39-0.50)	0.45 (0.39-0.51)	**0.45 (0.39-0.50)**	0.42 (0.37-0.47)
r_A	0.42 (-0.02-0.87)	0.35 (-0.39-1.00)	**0.36 (0.23-0.48)**	NA
r_D	NA	1.00 (-1.00-1.00)	**NA**	NA
r_H	0.42 (-0.02-0.87)	0.36 (0.23-0.50)	**0.36 (0.23-0.48)**	NA
r_C	-1.00 (-1.00-1.00)	NA	**NA**	0.24 (0.11-0.36)
r_E	0.73 (0.43-1.00)	0.74 (0.43-1.00)	**0.74 (0.45-1.00)**	0.75 (0.57-0.93)
Bivariate A	0.66 (-0.02-1.33)	0.51 (-0.58-1.59)	**0.59 (0.41-0.77)**	NA
Bivariate D	NA	0.10 (-1.05-1.24)	**NA**	NA
Bivariate H	0.66 (-0.02-1.33)	0.60 (0.41-0.80)	**0.59 (0.41-0.77)**	NA
Bivariate C	-0.06 (-0.64-0.51)	NA	**NA**	0.35 (0.18-0.52)
Bivariate E	0.41 (0.21-0.60)	0.40 (0.20-0.59)	**0.41 (0.23-0.59)**	0.65 (0.48-0.82)

AIC, Akaikes information criterion; BIC, Bayesian information criterion; T1D, type 1 diabetes; HT, Hashimoto's thyroiditis; A, additive genetics/narrow sense heritability; D, dominance deviations; H, broad sense heritability (A+D); C, shared environment; E, individually unique environment; ACE, ADE, AE, CE, models containing named sources of (co)variance; NA, not applicable; Bivariate A, explained phenotypic correlation by A sources of (co)variance (similar for D, H, C, and E).

Confidence intervals are of Wald-type, thus they may span outside the parameter space, but have been truncated at the parameter bound. **Bold** font indicates best fitting model according to AIC and BIC.

[a] Adjusted for sex and birth cohort.

Supplementary Table 5. Concordances and discordances for APS-3, i.e. type 1 diabetes and Hashimoto's thyroiditis combined.

	Concordant non-affected	Discordant	Concordant affected	Concordance rate	Tetrachoric correlation[a]
APS-3 monozygotic	17,977	14	4	0.36 (0.17-0.74)	0.88 (0.76-1.00)
APS-3 dizygotic	37,386	26	0	0.000 (NA)	-0.62 (-1.00-1.00)

APS-3, having both type 1 diabetes and Hashimoto's thyroiditis.
[a] Unadjusted.

Supplementary Table 6. Results for APS-3, i.e., type 1 diabetes and Hashimoto's combined, crude model.

	ACE	ADE	**AE**	CE
AIC	-220,829.15	-220,829.57	**-220,831.15**	-220,825.61
BIC	-1,209,536.00	-1,209,536.42	**-1,209,546.92**	-1,209,541.38
A	85% (71-98)	0% (0-0)	**85% (71-98)**	NA
D	NA	85% (72-99)	**NA**	NA
H	85% (71-98)	85% (72-99)	**85% (71-98)**	NA
C	0% (0-0)	NA	**NA**	74% (59-89)
E	15% (2-29)	15% (1-28)	**15% (2-29)**	26% (11-41)

AIC, Akaike's information criterion; BIC, Bayesian information criterion; A, additive genetics/narrow sense heritability; D, dominance deviations; H, broad sense heritability (A+D); C, shared environment; E, individually unique environment; ACE, ADE, AE, CE, models containing named sources of (co)variance; NA, not applicable.
Confidence intervals are of Wald-type, thus they may span outside the parameter space, when this happened the interval has been truncated at the parameter bound. **Bold** font indicates best fitting model according to AIC.

Supplementary Figure 1 Year of, and age at diagnosis.

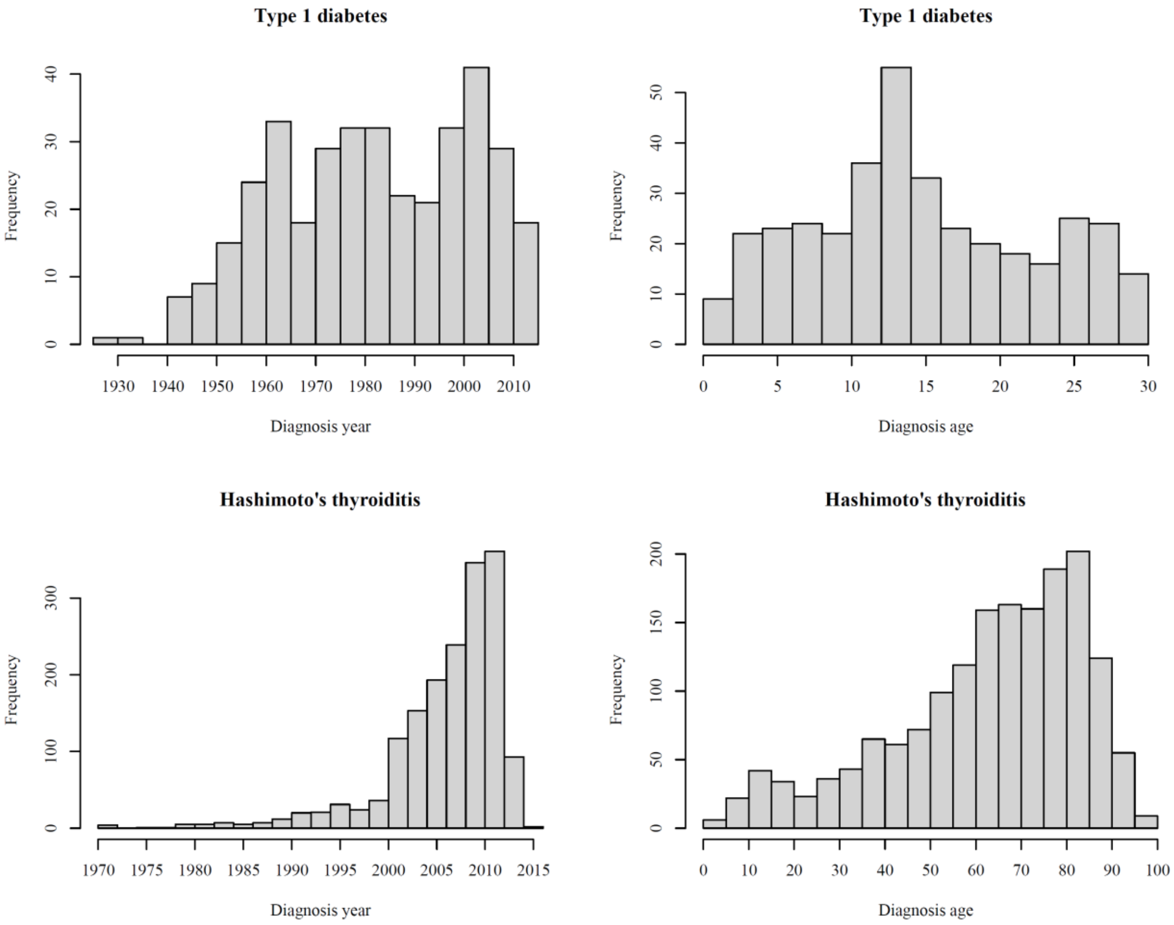

Supplement: Supplementary Material [file supplementary_material.pdf]
